# Mining prokaryotic genomes for unknown amino acids: a stop-codon-based approach

**DOI:** 10.1186/1471-2105-8-225

**Published:** 2007-06-28

**Authors:** Masashi Fujita, Hisaaki Mihara, Susumu Goto, Nobuyoshi Esaki, Minoru Kanehisa

**Affiliations:** 1 Institute for Chemical Research, Kyoto University, Gokasho, Uji, Kyoto 611-0011, Japan

## Abstract

**Background:**

Selenocysteine and pyrrolysine are the 21st and 22nd amino acids, which are genetically encoded by stop codons. Since a number of microbial genomes have been completely sequenced to date, it is tempting to ask whether the 23rd amino acid is left undiscovered in these genomes. Recently, a computational study addressed this question and reported that no tRNA gene for unknown amino acid was found in genome sequences available. However, performance of the tRNA prediction program on an unknown tRNA family, which may have atypical sequence and structure, is unclear, thereby rendering their result inconclusive. A protein-level study will provide independent insight into the novel amino acid.

**Results:**

Assuming that the 23rd amino acid is also encoded by a stop codon, we systematically predicted proteins that contain stop-codon-encoded amino acids from 191 prokaryotic genomes. Since our prediction method relies only on the conservation patterns of primary sequences, it also provides an opportunity to search novel selenoproteins and other readthrough proteins. It successfully recovered many of currently known selenoproteins and pyrrolysine proteins. However, no promising candidate for the 23rd amino acid was detected, and only one novel selenoprotein was predicted.

**Conclusion:**

Our result suggests that the unknown amino acid encoded by stop codons does not exist, or its phylogenetic distribution is rather limited, which is in agreement with the previous study on tRNA. The method described here can be used in future studies to explore novel readthrough events from complete genomes, which are rapidly growing.

## Background

Stop codon readthrough is a phenomenon in which the translation process does not terminate at a stop codon, and an amino acid is inserted there instead [1,2]. In some cases, the inserted amino acid is not one of the 20 amino acids but a noncanonical one. Two such amino acids have been discovered to date: selenocysteine [3,4] and pyrrolysine [5,6]. Because each of them have specialized tRNA genes for decoding and can be considered extensions of the standard genetic code, they are called the 21st and 22nd amino acids, respectively. Selenocysteine, the 21st amino acid, is encoded by stop codon UGA, and organisms that use selenocysteine have been found from all three domains of life. Its insertion into UGA is directed by SECIS (selenocysteine insertion sequence) elements, a stem-loop structure on the selenoprotein mRNA. Along with the progress of genome sequencing projects, computational prediction methods of selenocysteine-containing proteins (selenoproteins) have been developed by several research groups [7-10], and the repertoire of selenoproteins has been greatly expanded [11,12]. Pyrrolysine, the 22nd amino acid encoded by stop codon UAG, was recently discovered from a methanogenic archaea [5,6]. Currently, only methanogenic archaea of the order Methanosarcinales and one bacterium are considered to utilize pyrrolysine [13]. The limited phylogenetic distribution of pyrrolysine suggests that its incorporation into the genetic code of methanogen is relatively recent, and the insertion mechanism of a novel amino acid can evolve in a shorter period of time than anticipated.

This raises an interesting question: "Is there a 23rd amino acid?" If such an amino acid is discovered, it will deepen our understanding of the evolution and diversity of the genetic code. Because genome sequences of various prokaryotes are available today, there will be a chance to discover the novel amino acid via analysis of these genomes. Since both the 21st and 22nd amino acids are encoded by stop codons, the prime suspect is other stop codons (e.g. stop codon UAA), although the possibility of sense codons certainly remains. Using this clue, computational screening methods of the 23rd amino acid can be designed. Recently, Lobanov *et al*. addressed this problem by searching tRNAs with anticodons corresponding to stop codons [14]. They analyzed 146 prokaryotic genomes, but no likely tRNA of the novel amino acid was detected. They concluded that the 23rd amino acid would have a limited phylogenetic distribution, if it exists.

However, programs for tRNA identification are based on the features of known tRNAs and do not necessarily perform well on unknown ones. Actually, tRNA^Sec ^and tRNA^Pyl ^have unusual secondary structures [5,15] and often escape detection by programs without special consideration. Lobanov *et al*. thus developed a sensitive search method to deal with this problem, but they also admitted that it would fail to identify highly unusual tRNAs. There is another approach to searching for the 23rd amino acid. By enumerating ORFs that have an inframe stop codon from genomes and examining their evolutionary conservation, candidate proteins can be predicted. Because such an ORF-based study is independent from the tRNA analysis, it can either identify candidate organisms missed by the previous study or strengthen its negative conclusion.

Here we report a comprehensive analysis of prokaryotic ORFs that contain an inframe stop codon. Through enumeration of theoretical ORFs and inspection of their evolutionary conservation, candidates of readthrough proteins were predicted. They contained many of the known proteins with stop-codon-encoded amino acids, but almost no novel candidates were identified. Therefore, the unknown amino acid, if it is encoded by a stop codon, is unlikely to exist in the current databases of microbial genomes. The consequences for selenoproteins and other readthrough genes are also discussed.

## Results

### Basic ideas

**Figure 1:**
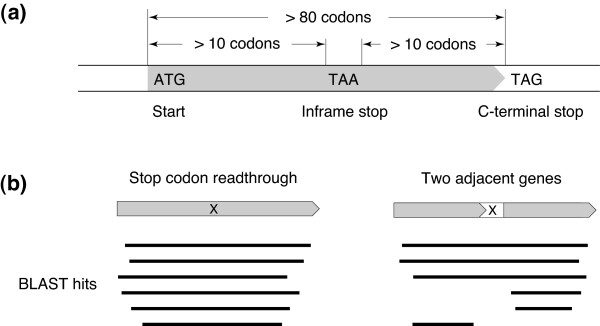
Basic ideas of the prediction method. **(a) **Schematic illustration of an interrupted ORF (iORF). **(b) **Readthrough genes can be distinguished from two adjacent genes based on the results of BLAST searches. Boxes denote iORFs, and × indicates the inframe stop codon. Shaded regions represent actual protein-coding regions. If an iORF codes a readthrough protein, BLAST hits from other organisms will cover the inframe stop codon. In contrast, if the iORF consists of two adjacent genes, many hits that do not cover the inframe stop codon will be found.

In this study, we focus on theoretical ORFs with one inframe stop codon, termed "interrupted ORFs" (iORFs) (Figure 1a). If we enumerate all iORFs from microbial genomes, most of the readthrough genes will be included in them. However, the vast majority of the enumerated iORFs will be biologically meaningless. To filter out such meaningless iORFs, we required the iORFs to have at least one homolog in other genomes, because evolutionary conservation of primary sequence is a strong indicator of functional importance. However, this condition is not sufficient, since two major problems remain: pseudogenes and two adjacent genes. The first problem is that even if an iORF has homologs in other species, it could be a pseudogene or a product of sequencing error. The second problem is that adjacent genes on the same reading frame may satisfy the condition of conserved iORFs. In particular, gene pairs within an operon are problematic because their gene arrangement is often conserved. If the intergenic distance between two genes in an operon happens to be a multiple of three, they look like a conserved readthrough gene.

To discriminate them from true readthrough genes, evolutionary information was exploited. In order to eliminate pseudogenes and sequencing errors, conservation of iORFs and their inframe stop codons was examined. Since pseudogenes are less conserved, and sequencing errors are relatively rare events, they will not have homologous iORFs in other species. Even if they do, the position or type (UAA, UAG or UGA) of their inframe stop codons will not coincide. In this way, they can be eliminated as candidates. A drawback of this criterion is that it limits the target of our study to readthrough genes conserved across two or more species. In other words, species-specific readthrough genes are not in the scope of this study.

To address the second problem, adjacent gene pairs were filtered out by examining boundaries of sequence alignments between iORFs and its homologs (Figure 1b). The stop-codon-encoded amino acids of prokaryotes are usually located inside domains, the units of evolutionary sequence conservation. Therefore, the aligned regions of readthrough proteins contain their inframe stop codon. Based on this observation, each iORF was required to have: (i) at least one homolog from other organisms that covers the inframe stop codon and (ii) no homolog that does not cover the stop codon. Note that, however, if the whole length of an iORF was used as a query sequence, this procedure will erroneously discard multidomain readthrough proteins. To avoid this problem, a partial sequence around the inframe stop codon was used as a query.

### Prediction procedure

**Figure 2:**
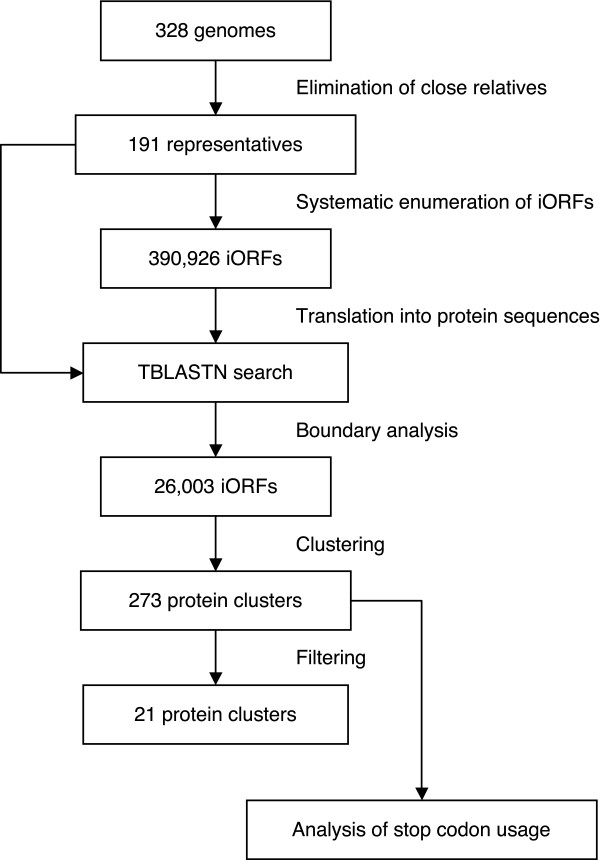
A flowchart of the prediction procedure. Several steps are omitted for simplicity. Detailed explanation is given in the text.

The prediction schema is shown in Figure 2. A total of 191 prokaryotes were analyzed in this study, of which 166 are bacteria and 25 are archaea. They were selected from 328 prokaryotes with completely sequenced genomes by excluding closely related species. From the genome sequences of the 191 organisms, all possible iORFs were enumerated. Two conditions were imposed on the geometry of the iORFs (Figure 1a). First, only iORFs longer than 80 codons were extracted. Secondly, margins between the inframe stop codon and both termini of the iORF must be longer than 10 codons. The total number of iORFs extracted under these conditions was 2,969,958. Next, iORFs that overlap RNA genes or protein-coding genes in different reading frames were discarded. This test significantly reduced the number of iORFs to 390,926.

As noted above, the target of this study is evolutionarily conserved iORFs. Thus, it was examined whether the iORFs have homologous regions in other genomes. The 390,926 iORFs were translated into amino acid sequences and subjected to TBLASTN [16] against the 191 genome sequences. Instead of the whole length of the amino acid sequence, a window of 101 residues centered at the inframe stop codon was used as a BLAST query. After the BLAST searches, iORFs that have at least one interspecific hit that contains the inframe stop codon were collected. Whether the codon aligned to the inframe stop codon is a nonsense codon or not was neglected at this stage. There were 94,690 iORFs that have interspecific hits. The result of the above homology searches was also used for the boundary analysis (Figure 1b). An iORF was discarded if there were any BLAST hits that do not cover the inframe stop codon. A total of 26,003 iORF satisfied the above criteria.

To examine intrafamily conservation of the inframe stop codons, these iORFs were clustered into protein families based on sequence similarity. After removal of singletons, 679 clusters with two or more members were obtained. A cluster was discarded unless all members of the cluster had the same type of inframe stop codons (UAA, UAG or UGA). The locations of the inframe stop codons were also required to be identical in the multiple sequence alignment of the cluster members. These conditions reduced the number of clusters to 273.

Manual inspection of these 273 clusters revealed that they still contain many false positives that are unrelated to stop-codon-encoded amino acids. Hence, three-step filtering procedures were applied to remove the false positives. Briefly, the first filter assesses protein-likeliness based on the signal of purifying selection, while the second and third filters try to remove adjacent gene pairs using the pattern of BLAST alignments (for details, see Materials and Methods). As a result of the filtering, the number of candidate clusters was reduced to 32. Through manual inspection of the BLAST alignments, 11 clusters were discarded because they are highly unlikely to code readthrough proteins.

### Known proteins in the predicted clusters

**Figure 3:**
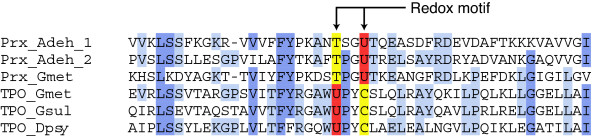
Selenoprotein families we failed to detect because of nonconserved location of stop codons. Selenocysteine residues of Peroxiredoxin-like protein families constitute homologous redox motifs (TXXU and UXXC), but their positions are different between two families. Columns are colored according to sequence conservation. Selenocysteine residues are shown in red, and the other residues in the redox motifs are shown in yellow. Prx; Peroxiredoxin, TPO; thiol:protein disulphide oxidereductase, Adeh; *Anaeromyxobacter dehalogenans*, Gmet; *Geobacter metallireducens*, Gsul; *G. sulfurreducens*, Dpsy; *Desulfotalea psychrophila*. The alignments were computed using ClustalW, and the figures were generated using Jalview.

The clusters predicted by our method are summarized in Table 1. Of the 21 clusters, 15 were known selenoproteins, and four were known pyrrolysine proteins. To assess the sensitivity of our method, the result was compared with a list of prokaryotic selenoproteins reported by Kryukov and Gladyshev [12]. Since our target is readthrough genes conserved across two or more species, such selenoprotein families were selected from their list. There were 15 families satisfying this criterion, but one family, proline reductase, was excluded because it was found in only one organism in our dataset. Of the 14 families, 11 were found in our prediction result. The three families we failed to find were SelW-like protein, peroxiredoxin and thiol:protein disulphide oxidoreductase. SelW-like protein was below the threshold of detection, because its stop codon is near the N-terminus and the amino acid sequences of its members are too divergent. The reason why the two other families were not detected is more complex. Since these two families are homologous, they were grouped into an identical cluster at the clustering stage of our method. However, the positions of selenocysteine were different between the two families (Figure 3). The cluster was thus discarded because of an apparent lack of stop codon conservation. To deal with a situation like this, a reexamination of the clustering threshold and subdivision of clusters will be required.

**Table 1:** Predicted clusters of readthrough proteins

Cluster description	Codon	Size	Example organism (locus)
**Selenocysteine**			
Formate dehydrogenase α subunit	TGA	45	Escherichia coli (b1474)
Selenide water dikinase	TGA	12	Haemophilus influenzae (HI0200m)
Glycine reductase complex selenoprotein A	TGA	6	Treponema denticola (TDE0745)
Glycine reductase complex selenoprotein B	TGA	6	Treponema denticola (TDE0078)
Heterodisulfide reductase subunit A	TGA	6	Methanococcus jannaschii (MJ1190m)
Coenzyme F420-reducing hydrogenase δ subunit	TGA	5	Methanococcus jannaschii (MJ1190a)
Formylmethanofuran dehydrogenase subunit B	TGA	4	Methanococcus jannaschii (MJ1194m)
Glutaredoxin-like	TGA	3	Carboxydothermus hydrogenoformans (CHY_0740)
Thioredoxin	TGA	3	Geobacter sulfurreducens (GSU3446)
Coenzyme F420-reducing hydrogenase α subunit	TGA	3	Methanococcus jannaschii (MJ0029)
HesB family	TGA	3	Desulfovibrio vulgaris (DVU_1382)
HesB family	TGA	2	Methanococcus maripaludis (MMP0252 + upstream)
Fe-S oxidoreductase	TGA	2	Desulfotalea psychrophila (DP1009)
DsbA-like	TGA	2	Desulfovibrio desulfuricans (Dde_1263 + upstream)
Periplasmic [NiFeSe] hydrogenase large subunit	TGA	2	Desulfovibrio vulgaris (DVU_1918)
**Pyrrolysine**			
Monomethylamine methyltransferase	TAG	7	Methanosarcina acetivorans (MA0144)
Dimethylamine methyltransferase	TAG	7	Methanosarcina acetivorans (MA0532)
Trimethylamine methyltransferase	TAG	6	Methanosarcina acetivorans (MA0528)
Transcriptional regulator, TetR family	TAG	2	Methanosarcina acetivorans (MA2902)
**Unknown**			
Cytochrome c family protein	TGA	2	Geobacter sulfurreducens (GSU2937 + GSU2936)
Hypothetical protein	TAG	2	Geobacter sulfurreducens (GSU2293 + downstream)

A plus sign in a locus indicates that the genomic coordinates of the iORF can be described by a concatenation of two genes or regions. For example, "GSU2293 + downstream" means that the iORF consists of the gene GSU2293 and its downstream sequence. HesB family was not clustered into one family, because their sequences were too short and diverged.

Of the four pyrrolysine proteins detected, three methylamine methyltransferases have been experimentally confirmed to contain pyrrolysine [6,17]. The rest is a cluster of TetR-like transcriptional regulators from *Methanosarcina acetivorans *and *M. barkeri*. Since the genome annotation of *M. acetivorans *describes this protein as a gene containing an inframe amber codon, we classified it as a 'known' candidate, although it is still unclear whether it really contains pyrrolysine. The genome annotation of *M. acetivorans *also includes several amber-containing genes that were absent from our prediction result. They are a methlycobamide:CoM methylase and four transposases [18]. The reason why they were not detected is that only one species in our dataset had an amber-containing form of these proteins. This is unavoidable because of the inability of our method to detect species-specific readthrough events. It is the price for reliably excluding pseudogenes and sequencing errors.

### Unknown candidates in the predicted clusters

**Figure 4:**
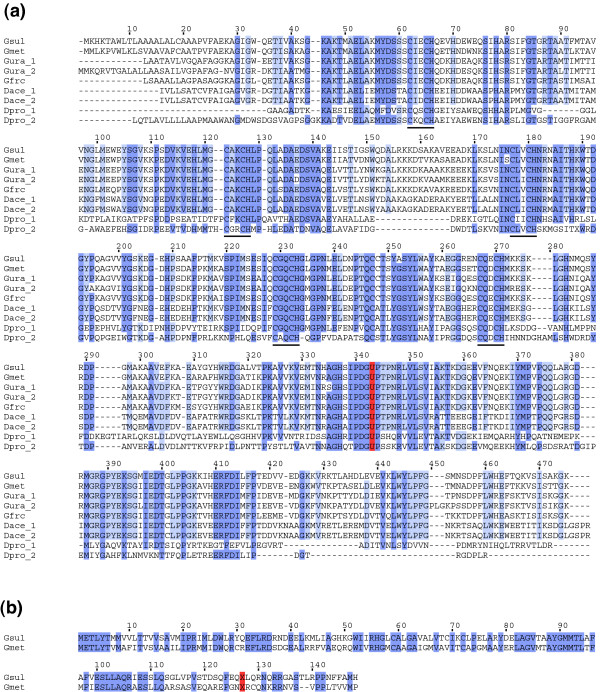
Multiple sequence alignments of novel candidate proteins. **(a) **A selenoprotein candidate from *Geobacter sulfurreducens *and its homologs. The possible selenocysteine residues are shown in red, and putative heme-binding motifs are underlined. Note that sequence conservation near the selenocysteine is comparable to that of the N-terminal cytochrome domain. A protein Dpro_2 contains yet another inframe stop codon (TAG) at the column 189. It will be either a sequencing error or a pseudogene. Gsul; *G. sulfurreducens*, Gmet; *G. metallireducens*, Gura; *G. uraniumreducens*, Gfrc; *Geobacter *sp. FRC-32, Dace; *Desulfuromonas acetoxidans*, Dpro; *Delta proteobacterium *MLMS-1. **(b) **Hypothetical proteins from *Geobacter *species. The inframe stop codons (TAG) are shown in red. This cluster is probably an artifact of close phylogenetic relationship.

The successful detection of many known proteins is encouraging, because our method relies only on general properties of proteins that contain stop-codon-encoded amino acids, but not on specific features of selenocysteine or pyrrolysine. Therefore, unknown clusters in our candidates have possibilities for the 23rd amino acid or novel readthrough proteins. There were two such clusters (Table 1). The first cluster is comprised of c-type cytochromes from δ-proteobacteria *Geobacter sulfurreducens *and *G. metallireducens*. The N-terminal part of the sequence contains five CXXCH heme-biding motifs, while the C-terminal part has no similarity with any characterized proteins. Homology search against unfinished microbial genomes identified seven homologous proteins from four other δ-proteobacteria species. Multiple sequence alignment of these sequences is shown in Figure 4a.

We expect that this cluster may represent a novel selenoprotein family. This is because the inframe stop codons of these proteins are exclusively TGA, and all of the above organisms possess selenocysteine insertion machinery (data not shown). High conservation of residues near the inframe stop codon also suggests the importance of this region. If they are true selenoproteins, this protein family becomes a rare instance of selenoprotein that lacks non-selenocysteine homologs. However, computational analysis of sequences immediately downstream of the inframe stop codons failed to identify SECIS elements, which is a hallmark of selenocysteine-containing genes. Therefore, yet another possibility is that they are a highly conserved operon. An experimental verification is necessary to distinguish these two possibilities.

The second cluster consists of two hypothetical proteins, again from *G. sulfurreducens *and *G. metallireducens *(Figure 4b). In contrast to the first cluster, no homolog was identified from other species. This cluster is probably a false positive and not readthrough proteins. This is because the residues near the inframe stop codons are poorly conserved. Moreover, the C-terminal extensions are quite short (about 20 aa). The sequence conservation in this region can be easily explained by the close phylogenetic relationship between the two species. In summary, although a possible selenoprotein was newly identified, there was no promising candidate for an unknown amino acid encoded by a stop codon.

### Stop codon usage in the pre-filtering clusters

The above negative result could be explained if the filtering process, which is the final step of the prediction method (Figure 2), was too strict. Although the raw output of the search for evolutionarily conserved iORFs was 273 clusters, most of them were discarded at the subsequent filtering stage. Because we have no *a priori *knowledge about the 23rd amino acid, cutoff thresholds for the filtering procedures were determined based on the known readthrough proteins. This is practically indispensable for objective classification of candidates, but there is no guarantee that unknown proteins with the 23rd amino acid will score higher than the thresholds.

**Figure 5:**
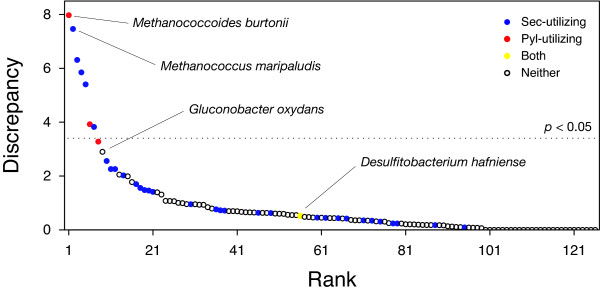
Discrepancies of stop codon usages between the inframe and C-terminal stop codons. The inframe stop codon usage is taken from the pre-filtering clusters, and the C-terminal usage is computed based on the annotated proteins of the organism. Red circle: an organism with pyrrolysine, blue; selenocysteine, yellow; both pyrrolysine and selenocysteine, white; neither pyrrolysine nor selenocysteine. The organisms are ordered by their discrepancy scores. The discrepancy score is the negative logarithm of a *p*-value of Fisher's exact test. The dotted line indicates significance level 0.05 after a correction for multiple testing.

To explore whether a number of good candidates lie below the thresholds, the 273 clusters were analyzed in a way independent from filtering. If an organism has many readthrough proteins, proteins from the organism will frequently appear in the 273 clusters. Moreover, relative usage of the inframe stop codons will deviate from that of usual termination signals in the proteome. Figure 5 shows the discrepancies between relative usage of the inframe and C-terminal stop codons of 127 organisms in the pre-filtering clusters. Only seven organisms had statistically significant discrepancies (*P *< 0.05), and all of them are known to utilize selenocysteine or pyrrolysine.

When top ten organisms were examined, only *Gluconobacter oxydans *was an organism not known to have stop-codon-encoded amino acids. An inspection of the *G. oxydans *iORFs in the 273 clusters revealed that their inframe stop codons are dominated by TAA, but all of them belong to a single protein cluster associated with transposable elements. Because it seems unlikely that an insertion system of novel amino acid evolves solely for transposable elements, this organism cannot be considered as a good candidate of the 23rd amino acid. Sensitivity of this test is not high because many organisms that utilize selenocysteine were below the defined threshold. However, the result agrees with the filtering-dependent analysis that no candidate of the novel stop-codon-encoded amino acid is detectable in the current dataset.

## Discussion

As the number of completely sequenced genomes increases, several research groups started to predict proteins that contain stop-codon-encoded amino acids through computational analyses. Most of them are aimed at identification of selenoproteins, reflecting concerns from the scientific community and accumulated knowledge on selenocysteine. In order to improve prediction specificity, they have fully exploited the known features of selenocysteine, such as the SECIS elements or cysteine homologs, which have cysteine in place of selenocysteine. However, since the target of this study is the 23rd amino acid, and there is no *a priori *knowledge, only general properties of stop-codon-encoded amino acids can be used for prediction. Such general-purpose algorithms have also been developed to date. The method of Chaudhuri and Yeates [10] extracts iORFs from microbial genomes and analyzes sequence conservation around the inframe stop codon. Their method is thus similar to ours and applicable to both selenocysteine and pyrrolysine. Perrodou *et al*. [19] constructed a database of predicted recoding events in microbes. Their method is applicable not only to stop codon readthrough but also to frameshift.

However, both of them did not apply their methods to search for novel amino acids. Therefore, the question of the 23rd amino acid has not been investigated from the viewpoint of coding sequences. Additionally, the previous methods cannot effectively discriminate pseudogenes from readthrough genes. For instance, Chaudhuri and Yeates reported a homolog of cobalamin biosynthesis protein CobN as a novel candidate of pyrrolysine protein. However, the gene is probably a pseudogene because it contains an inframe TAA codon in addition to the TAG codon, and only one species seems to have the amber-containing form of the gene.

The previous methods also assume that proteins with stop-codon-encoded amino acids will have non-readthrough homologs (i.e., homologous proteins that do not have inframe stop codons). However, that is not necessarily true. For example, pyrrolysine-containing monomethylamine methyltransferases adopt TIM barrel fold [6], but their primary sequences do not exhibit detectable similarity to other TIM barrel proteins because of evolutionary divergence. Dimethylamine methyltransferases also lack non-readthrough homologs. Yet another example is glycine reductase selenoprotein A. Only the selenocysteine-containing form of the enzyme is currently known [20]. Therefore, it is important not to assume non-readthrough homologs for exploring novel candidates. If any non-readthrough homologs are registered in public sequence databases, a careful annotation process of a newly sequenced genome will be able to detect readthrough genes, even though they may be annotated as pseudogenes. However, if all members of a gene family have stop codon readthrough, correct annotation of their gene structure will be extremely difficult, and all of them will be split into two distinct genes.

The method reported here is unique in that it does not assume non-readthrough homologs. Using this method, a systematic screening of the 23rd amino acid and other readthrough genes was carried out. Many of the currently known selenoproteins and pyrrolysine proteins were recovered, indicating the effectiveness of this approach. In particular, successful detection of pyrrolysine-containing methyltransferases and selenoprotein A should be noted. However, almost no novel candidates for readthrough genes were predicted. What can be concluded from this result? The most likely explanation is that the 23rd amino acid does not exist, or its distribution on the tree of life is rather limited. Although a broad spectrum of taxonomic groups has been subjected to genome sequencing, the genomes of most microbial species on the earth have yet to be determined. The unknown amino acid may be used by these species. Alternatively, only one organism in our dataset may have the 23rd amino acid. This is because our method is limited to readthrough genes conserved across two or more species. If the novel amino acid appears in younger, non-conserved sequences, our technique will miss them. In either case, the distribution of the 23rd amino acid will be significantly narrower than that of selenocysteine, which has scattered but wide distribution [21]. This conclusion coincides with and strengthens that of the previous research on tRNA [14].

Yet another possibility is that the 23rd amino acid exists but is not encoded by stop codons. It is well known that the genetic code varies in several organisms [22]. Thus, certain organisms may use one of the sense codons for the novel amino acid. Because codons for most amino acids are degenerate, redefinition of one of them is feasible. However, that possibility is beyond the scope of this study and is left as an open problem. Bioinformatics analysis of unusual tRNA genes and codon usage may provide insights into this problem.

In addition to the 23rd amino acid, our method can simultaneously explore selenoproteins and other readthrough proteins. A common assumption in microbial selenoprotein predictions is that selenoproteins will have cysteine homologs. Zhang *et al*. [20] examined the validity of this assumption using a SECIS-based method and concluded that selenoproteins without cysteine homologs will be extremely rare. Our method can reassess this assumption in a SECIS-independent way. Such selenoproteins identified through our screening of nearly 200 microbial genomes were selenoprotein A and only one uncertain candidate. Therefore, selenoproteins that lack cysteine homologs will be scarce, as previously reported.

Other readthrough proteins with canonical amino acids (i.e., proteins that have canonical amino acids at their inframe stop codons) are quite rare in prokaryotes [1]. The result reported here is in agreement, but it is not conclusive. This is because our method assumes that stop-codon-encoded amino acid is located inside a domain, but it is unclear whether it holds true in prokaryotic readthrough with canonical amino acids. At least, only one experimentally-confirmed example from a pathogenic strain of *Escherichia coli *[23], whose genome is not yet determined, does not obey this rule. What can be concluded from our result is that this type of readthrough will be located outside of domains, such as a linker between two domains. Such a stop codon may behave as a switch that regulates production of short and long isoforms from a single mRNA, as in readthrough genes from viruses [24].

## Conclusion

To explore the possibility of a 23rd amino acid, ORFs in prokaryotic genomes were investigated in a comprehensive way. Although many of the currently known selenoproteins and pyrrolysine proteins were successfully detected, no candidate for the 23rd amino acid was discovered. Therefore, if such an amino acid exists, it will have limited distribution in the tree of life. Alternatively, it may be encoded by one of the sense codons. From the viewpoint of selenoprotein prediction, the sensitivity of our method was lower than an existing method. However, our method has several unique features. It is applicable to general readthrough genes and rigorously excludes pseudogenes and sequencing errors. Moreover, it does not assume the occurrence of non-readthrough homologs in the public databases. It will help in identification of novel readthrough genes from the rapidly expanding collection of complete microbial genomes.

## Methods

### Enumeration of iORFs from prokaryotic genomes

A total of 328 complete genome sequences of prokaryotes were downloaded from the KEGG FTP site [25] in April 2006. From them, 191 representative organisms were selected by excluding close relatives. The threshold was set to average sequence identity 90% of two house-keeping genes, DNA polymerase III α subunit and alanyl-tRNA synthetase. From these 191 genomes, iORFs longer than 80 codons were enumerated using inhouse software, which is available from the author's web site [26]. Both upstream and downstream regions of its inframe stop codon were required to be longer than 10 codons. Two stop codons of an iORF (i.e. the inframe and C-terminal stops) can be any combination of canonical stop codons (TAA, TAG, TGA). However, for Mycoplasma, only TAA and TAG were used. Three codons ATG, TTG and GTG were allowed to be start signals.

The iORFs of each organism were compared with protein-coding genes of the organism using BLASTX. If an iORF matched any protein-coding genes (*E*-value < 10^-3^) and their reading frames did not coincide, the iORF was discarded. Similarly, iORFs were compared with RNA genes using BLASTN, and those matched with the RNAs were removed. Remaining iORFs were translated into amino acid sequences. We translated all three types of nonsense codons into the one-letter code U, so as to simplify visual inspection of sequence alignments. Although the code U is usually for selenocysteine, it will be harmless because U is automatically converted into × inside the BLAST programs.

### Construction of clusters of conserved iORFs

To examine evolutionary conservation of the iORFs, a window of 101 residues around the inframe stop codon was extracted and subjected to TBLASTN searches against the above 191 genome sequences. If there were any hits (*E*-value < 0.01) in other organisms, and if the hit includes 10 upstream and 10 downstream residues of the inframe stop codon, then the iORF was retained. However, if there were any hits (*E*-value < 10^-5^) that did not cover the inframe stop codon, the iORF was discarded. Eligible iORFs were then clustered using BLASTCLUST with score density 0.5 and minimum length coverage 0.6. After removing singleton clusters, multiple sequence alignments of the remaining clusters were computed using MAFFT [27] with the L-INS-i option. Subsequently, conservation of the inframe stop codons in each cluster was examined. If the location or type of stop codons was not identical, the cluster was discarded.

### Three-step filtering of the candidate clusters

The first filter examines protein-likeliness of the iORFs. This filter is mainly designed to remove conserved non-coding sequences (CNS) immediately downstream of non-readthrough genes. If we measure purifying selection for amino acid sequences by the ratio of nonsynonymous to synonymous substitution rates (*dN/dS*), a protein with a stop-codon-encoded amino acid will indicate the sign of selection, while CNS will not. The *dN/dS *was calculated for each of the two parts flanking the inframe stop codon in an iORF using codeml program in the PAML package [28]. Statistical significance was estimated by likelihood ratio test [29]. The observed alignment was fitted to two distinct substitution models, one of which estimates *dN/dS *from the data, and the other fixes it to 1.0. Let *l*_free _and *l*_fix _denote log likelihood of these models. Then, 2Δ*l *= 2(*l*_free _– *l*_fix_) approximately follows the χ^2 ^distribution with one degree of freedom. If *dN/dS *was less than 1.0, and the statistics 2Δ*l *was larger than a threshold, we regard it as a sign of purifying selection. In this study, the threshold was set to 5.0 (corresponds to *P *< 0.025) so that the known readthrough proteins score higher than the threshold. For each of the above clusters, an all-against-all comparison of cluster members was performed. If any pair exhibits such signals in both the N- and C-terminal parts, the cluster was retained.

Even if both the upstream and downstream regions of the inframe stop codon code proteins, they may be two adjacent genes instead of a readthrough protein. The second and third filtering processes remove such genes based on BLAST alignment patterns. Although the boundary analysis applied previously has the same goal (Figure 1b), some gene pairs escaped elimination. To enhance sensitivity of the filters, the whole length of an iORF was used as a BLAST query instead of the partial sequence, and the size of the BLAST database was increased from the 191 nonredundant genomes to the 328 complete genomes in GenomeNet and 246 draft genome sequences downloaded from GenBank in May 2006.

The second filter inspects synteny of iORFs. If the N- and C-terminal parts of an iORF have distinct but closely arranged BLAST hits in other genomes, it strongly suggests the iORF is actually two adjacent genes. Translated sequences of iORFs in the pre-filtering clusters were subjected to TBLASTN searches against the genome database. If both the best hits of the N- and C-terminal parts are statistically significant (*E*-value < 10^-5^), and distance between them is less than 1 kbp, we call these hits 'syntenic hits'. If any syntenic hits with non-coinciding reading frames were found, the cluster was removed.

The third filter uses co-occurrence of residues around the inframe stop codon as another source of information for screening stop codon readthrough. Suppose a window of 21 residues centered at the inframe stop codon. In prokaryotes, most stop-codon-encoded amino acids are located inside a domain, the unit of evolutionary sequence conservation. Therefore, in an ideal situation the presence or absence of the 21 residues in alignments will be synchronized. In contrast, if the iORF is actually two adjacent genes, then upstream and downstream residues of the stop codon will appear separately in many alignments. We defined a co-occurrence matrix as a 21 × 21 matrix whose (*i*,*j*)-th element represents how often residue *i *and *j *appeared simultaneously in *N *alignments. The matrix elements were subsequently normalized to the number of alignments *N*. By definition, the more often the upstream and downstream residues of the inframe stop codon co-occur in the alignments, the higher the density in the upper right quarter of the matrix. If average density in the quarter was lower than 0.85, the cluster was filtered out.

### Stop codon usage

For each organism, its iORFs were extracted from the pre-filtering clusters, and codon usage at the inframe stop positions was counted. Codon usage at the C-terminal stop codons in its proteome was also computed using data of coding sequences downloaded from KEGG GENES [25]. These data were combined into a 3 × 2 matrix, and Fisher's exact test was applied. The *p*-value was corrected for multiple testing using the Bonferroni correction because there were 127 organisms in the pre-filtering clusters.

## Authors' contributions

MF developed the method and drafted the manuscript. HM conceived and coordinated the study. All authors contributed to the writing, read and approved the final manuscript.

## References

[B1] Namy O, Rousset JP, Napthine S, Brierley I (2004). Reprogrammed genetic decoding in cellular gene expression. Mol Cell.

[B2] Cobucci-Ponzano B, Rossi M, Moracci M (2005). Recoding in archaea. Mol Microbiol.

[B3] Stadtman TC (1996). Selenocysteine. Annu Rev Biochem.

[B4] Hatfield DL, Gladyshev VN (2002). How selenium has altered our understanding of the genetic code. Mol Cell Biol.

[B5] Srinivasan G, James CM, Krzycki JA (2002). Pyrrolysine encoded by UAG in Archaea: charging of a UAG-decoding specialized tRNA. Science.

[B6] Hao B, Gong W, Ferguson TK, James CM, Krzycki JA, Chan MK (2002). A new UAG-encoded residue in the structure of a methanogen methyltransferase. Science.

[B7] Lescure A, Gautheret D, Carbon P, Krol A (1999). Novel selenoproteins identified in silico and in vivo by using a conserved RNA structural motif. J Biol Chem.

[B8] Kryukov GV, Kryukov VM, Gladyshev VN (1999). New mammalian selenocysteine-containing proteins identified with an algorithm that searches for selenocysteine insertion sequence elements. J Biol Chem.

[B9] Castellano S, Morozova N, Morey M, Berry MJ, Serras F, Corominas M, Guigó R (2001). In silico identification of novel selenoproteins in the Drosophila melanogaster genome. EMBO Rep.

[B10] Chaudhuri BN, Yeates TO (2005). A computational method to predict genetically encoded rare amino acids in proteins. Genome Biol.

[B11] Kryukov GV, Castellano S, Novoselov SV, Lobanov AV, Zehtab O, Guigó R, Gladyshev VN (2003). Characterization of mammalian selenoproteomes. Science.

[B12] Kryukov GV, Gladyshev VN (2004). The prokaryotic selenoproteome. EMBO Rep.

[B13] Zhang Y, Baranov PV, Atkins JF, Gladyshev VN (2005). Pyrrolysine and selenocysteine use dissimilar decoding strategies. J Biol Chem.

[B14] Lobanov AV, Kryukov GV, Hatfield DL, Gladyshev VN (2006). Is there a twenty third amino acid in the genetic code?. Trends Genet.

[B15] Commans S, Böck A (1999). Selenocysteine inserting tRNAs: an overview. FEMS Microbiol Rev.

[B16] Altschul SF, Madden TL, Schäffer AA, Zhang J, Zhang Z, Miller W, Lipman DJ (1997). Gapped BLAST and PSI-BLAST: a new generation of protein database search programs. Nucleic Acids Res.

[B17] Soares JA, Zhang L, Pitsch RL, Kleinholz NM, Jones RB, Wolff JJ, Amster J, Green-Church KB, Krzycki JA (2005). The residue mass of L-pyrrolysine in three distinct methylamine methyltransferases. J Biol Chem.

[B18] Galagan JE, Nusbaum C, Roy A, Endrizzi MG, Macdonald P, FitzHugh W, Calvo S, Engels R, Smirnov S, Atnoor D, Brown A, Allen N, Naylor J, Stange-Thomann N, DeArellano K, Johnson R, Linton L, McEwan P, McKernan K, Talamas J, Tirrell A, Ye W, Zimmer A, Barber RD, Cann I, Graham DE, Grahame DA, Guss AM, Hedderich R, Ingram-Smith C, Kuettner HC, Krzycki JA, Leigh JA, Li W, Liu J, Mukhopadhyay B, Reeve JN, Smith K, Springer TA, Umayam LA, White O, White RH, Conway de Macario E, Ferry JG, Jarrell KF, Jing H, Macario AJ, Paulsen I, Pritchett M, Sowers KR, Swanson RV, Zinder SH, Lander E, Metcalf WW, Birren B (2002). The genome of M. acetivorans reveals extensive metabolic and physiological diversity. Genome Res.

[B19] Perrodou E, Deshayes C, Muller J, Schaeffer C, Van Dorsselaer A, Ripp R, Poch O, Reyrat JM, Lecompte O (2006). ICDS database: interrupted CoDing sequences in prokaryotic genomes. Nucleic Acids Res.

[B20] Zhang Y, Gladyshev VN (2005). An algorithm for identification of bacterial selenocysteine insertion sequence elements and selenoprotein genes. Bioinformatics.

[B21] Romero H, Zhang Y, Gladyshev VN, Salinas G (2005). Evolution of selenium utilization traits. Genome Biol.

[B22] Knight RD, Freeland SJ, Landweber LF (2001). Rewiring the keyboard: evolvability of the genetic code. Nat Rev Genet.

[B23] Jalajakumari MB, Thomas CJ, Halter R, Manning PA (1989). Genes for biosynthesis and assembly of CS3 pili of CFA/II enterotoxigenic Escherichia coli: novel regulation of pilus production by bypassing an amber codon. Mol Microbiol.

[B24] Beier H, Grimm M (2001). Misreading of termination codons in eukaryotes by natural nonsense suppressor tRNAs. Nucleic Acids Res.

[B25] Kanehisa M, Goto S, Hattori M, Aoki-Kinoshita KF, Itoh M, Kawashima S, Katayama T, Araki M, Hirakawa M (2006). From genomics to chemical genomics: new developments in KEGG. Nucleic Acids Res.

[B26] RTFINDER. http://web.kuicr.kyoto-u.ac.jp/supp/fujita/rtfinder/.

[B27] Katoh K, Kuma K, Toh H, Miyata T (2005). MAFFT version 5: improvement in accuracy of multiple sequence alignment. Nucleic Acids Res.

[B28] Yang Z (1997). PAML: a program package for phylogenetic analysis by maximum likelihood. Comput Appl Biosci.

[B29] Yang Z, Bielawski JP (2000). Statistical methods for detecting molecular adaptation. Trends Ecol Evol.

